# Nucleosome structure incorporated histone acetylation site prediction in *arabidopsis thaliana*

**DOI:** 10.1186/1471-2164-11-S2-S7

**Published:** 2010-11-02

**Authors:** Chen Zhao, Hui Liu, Jiang Li, Youping Deng, Tieliu Shi

**Affiliations:** 1 Center for Bioinformatics and Computational Biology, and The Institute of Biomedical Sciences, School of Life Science, East China Normal University, 500 Dongchuan Road, Shanghai 200241, China; 2 Department of Biological Sciences, University of Southern Mississippi, Hattiesburg, MS-39406, USA; 3 Shanghai Information Center for Life Sciences, Chinese Academy of Sciences, Shanghai, China, 200031

## Abstract

**Abstract:**

**Background:**

Acetylation is a crucial post-translational modification for histones, and plays a key role in gene expression regulation. Due to limited data and lack of a clear acetylation consensus sequence, a few researches have focused on prediction of lysine acetylation sites. Several systematic prediction studies have been conducted for human and yeast, but less for *Arabidopsis thaliana*.

**Results:**

Concerning the insufficient observation on acetylation site, we analyzed contributions of the peptide-alignment-based distance definition and 3D structure factors in acetylation prediction. We found that traditional structure contributes little to acetylation site prediction. Identified acetylation sites of histones in *Arabidopsis thaliana* are conserved and cross predictable with that of human by peptide based methods. However, the predicted specificity is overestimated, because of the existence of non-observed acetylable site. Here, by performing a complete exploration on the factors that affect the acetylability of lysines in histones, we focused on the relative position of lysine at nucleosome level, and defined a new structure feature to promote the performance in predicting the acetylability of all the histone lysines in *A. thaliana*.

**Conclusion:**

We found a new spacial correlated acetylation factor, and defined a ε-N spacial location based feature, which contains five core spacial ellipsoid wired areas. By incorporating the new feature, the performance of predicting the acetylability of all the histone lysines in *A. Thaliana* was promoted, in which the previous mispredicted acetylable lysines were corrected by comparing to the peptide-based prediction.

## Introduction

Acetylation is a crucial post-translational modification for histones, and plays a key role in gene expression regulation [1]. Histone acetylation has the capacity to destabilize the chromatin polymer by charging neutralization of the basic lysine residue potentially harboring structural consequences for higher-order chromatin structures [2]. If a histone lysine is acetylable, it is reasonable to assume the existence of the function that affects the chromatin structure. Lysine acetylation in histones is regulated by a highly balanced enzyme system that contains lysine acetyltransferases (KATs) and histone deacetylases (HDACs) [3], and structural studies show that KAT domains coupled with peptide substrates typically do not exceed 14-20 amino acids (aa) in length[4].

There are two types of methods to predict protein acetylation modification, which are termed as N-terminal Acetylation Prediction and N^ε^-acetylation Prediction. Many recent acetylation prediction methods were proposed to analyze N^ε^-acetylation, such as PAIL[5] and PredMod [6]. Without distinguishing histone or non-histone and acetyltransferase types, PAIL method collects the training set by "acetylation lysine" keywords search and manually experimental verified filter. Whereas, PredMod focuses on the major human core histones bearing a total of 56 lysines, for the wealth of information known about their PTM patterns, well-developed purification and analytical detection methods. Almost, all of the previously prediction methods are based only on the flanking peptide sequence, even through commented the structure factors, such as solvent- accessible surface area, as additional or less-sense features.

Almost all the prediction methods are built on the data in human and yeast for their well-studied experimental data accumulation, but there are still lack of systematic analysis on *Arabidopsis thaliana*. Furthermore, the widely used MS approach has limited detection and sensitivity capabilities, and is unable to recognize peptides that are acetylated at only low abundance.

Core histones are highly conserved proteins, and there are very few differences for the amino acid sequences of the histone proteins between different species. Linker histone usually has more than one form within a species and is also less conserved than the core histones. Some variant forms appear in a few components of the major classes, they share amino acid sequence homology and have a core structural similarity to a specific class of major histones but also have their own features that distinct themselves from the major histones. These minor histones usually carry out specific functions of the chromatin metabolism. For example, histone H3.3 is associated with the body of actively transcribed genes[7]

Several important researches involved in distinguishing the post-translational modification patterns of histones in Arabidopsis have been published recently. Zhang et al have explored the core histone post-translational modification patterns in Arabidopsis thaliana [8], while Bergmuller et al. characterized post-translational modifications of histone H2B-variants isolated from Arabidopsis thaliana[9]. Those data provide a valuable resource to further explore the histone modification systematically.

Based on the association between structure and functional mechanism, the structural studies of KAT peptide substrates, and highly conservation of the histione, we performed an extensive exploration on the factors that affect the acetylability of lysines in histone, and predicted the acetylation sites through both human and *A. thaliana* experimental data. We firstly focused on the relative position of lysine at nucleosome level, and defined a new structure feature. Subsequently, we adopted the new feature to promote the predicted performance.

## Results

### Conservation of histones between Human and Arabidopsis

Histone H3 is highly conserved between Human and Arabidopsis with 97.8% identity, different only in the positions of 31, 87 and 90 amino acids, which is even with the similar identity level as that among the different isoforms within Arabidopsis. Histone H4 is also highly conserved between Human and Arabidopsis with 98.0% identity, different in the sites of 60 and 72. The identity of Histone H2B is 80.0% between human and Arabidopsis, but less conserved in the N- terminus, where the known acetylated site is localized. The acetylation information of histone H2B.C of human is used in a direct conservation-mapping mode in the prediction. Because of the lack of data about acetylation site and much more variants in histone H2A, the acetylation pattern of which is un-predictable by sequence conservation directly.

### Human acetylation based prediction

#### Known acetylation comparison between Human and Arabidopsis

In Arabidopsis, there are 13, 12, 12, 22, 16, 21 lysines in Histone H2A.1, H2A.2, H2A.3, H2A.4, H2A.5 and H2A.6, respectively, only two sites, K5 and K144 in H2A, have been verified for acetylation[8]. For histone H2B, there are 11 variants, but only H2B.10, H2B.6, H2B.7 have the acetylation information. Therefore, we considered those three variants in our prediction according to the filter principle (see methods). There are 29, 32 and 32 lysines in H2B.10, H2B.6, H2B.7, respectively, seven, six and six lysines of them were verified to be acetylatable. Similarly, both of histone H3.2 and H3.3 contain 14 lysines, seven lysines in each of them have shown to be acetylable. The least lysines exist in histone H4, and 50 percent of the total 10 lysines have been identified to be acetylated by our data collection (see methods). Meanwhile, text mining shows that only a few lysines (1/16 and 1/5) in H2A and H2B have been detected to be acetylated. Compared with human histone acetylation, identified acetylatable lysines of H2B are significantly less in Arabidopsis by Fisher Exact Test with p value less than 0.01.

#### Training set insufficient in Arabidopsis

The key hypothesis of flanking peptide based prediction methods is the structure recognization. Here, such type of methods focuses on short stretches of amino acid N- and C-terminus, because structural studies of published KAT domains coupled with peptide substrates typically do not exceed 14-20 aa in length [4,10]. Thus, the acetylatability of adjacent lysines is very interesting, and obviously has an effect on the peptide-based methods. Here, we take three as a distance to define the adjacent lysines. In the human training dataset (HTDS), there are 9 adjacent lysine pairs and eight of them have the same acetylatability with 89 identity, while only 53.0% identity remains in Arabidopsis training dataset (ATDS). Fisher's Exact Test shows that the relationship between adjacency and acetylability is different between the two training sets. By accepting the key hypothesis, the ATDS seems to be insufficient. Therefore, the highly conserved and well explored human acetylation data are adopted for the prediction in Arabidopsis.

### Human acetylation based prediction

#### Length normalization provide slightly data mining effect

The prediction method used here is mainly based on sequence similarity, as described in PreMod [6]. To construct a more stringent and theorizational model, several critical factors, including peptide length, lysine position and histone correlation, were considered.

To be consistent with the key hypothesis that acetylation is based on the structure reorganization of the lysine's flanking peptide, raw distance is defined as the local alignment score with BLOSUM 62 matrix and Smith-Waterman algorithm[11], and length normalized distance is equal to raw distance divided by the minimized length of the pair-wise alignment peptides. The hierarchical cluster dendrogram was constructed based on raw distance and length normalized distance. Cutting the dendrogram at a given height will split the tree into seperated classes (sub-classes) at a selected distance, the greater the number of the height, the less the amount of the seperated classes. Because the neighbour lysines are always with the same acetylation status, in such case, the number of the corresponding peptides was reduced to one according to PreMod. For the semi-supervised dataset with unobserved acetylation site labeled as N class, 'complete' hierarchical method and information gain ratio [12], are applied to construct and estimate the performance to balance the effect of minimizing sub-classes and maximizing accuracy, where 'complete' hierarchical method can minimize sub-classes and information gain ratio can maximize accuracy[12]. Comparing to the raw distance, length normalized distance reduces the sub-classes from 25 to 24 slightly, with the same information gain ratio 0.589 at the heights of 0.381 and 0.364 respectively, based on the hierarchical cluster dendrogram. As a visual data mining method, hierarchical cluster was used to summarize some chaos single sample into understandable patterns with different sub-classes. Whereas, to test whether such a method can cluster samples into simpler sub-classes, permutation method was employed to construct a significant level of random acetylation status labels. By 10000 permutations, p values of the Null hypothesis, that the number of sub-classes can be less than 25 or 24 by chance are 0.030 or 0.032, respectively. Furthermore, a 1000 time-permutation on normalization was also performed to test the distance, the length normalization effect is less significant with permutation p value as 0.27. The result showed that hierarchical cluster can group the peptides efficiently, but length normalization had less effect on the cluster result.

#### Human peptide acetylation based prediction performance

Two level prediction cutoffs were defined here. Because the raw tree with human peptide acetylation was built by complete hierarchical cluster method, adding any given different samples to the tree is unable to change the between-subclass relationships of the previous samples at any given height. The hierarchical structure was maintained with the high cutoff 0.364, and only the sites that are the children of the known human subclasses in the full cluster tree were considered to be predictable in acetylation prediction in Arabidopsis. Restricted to this cutoff, only 33 highly conserved sites in Arabidopsis were predicted, of which 12 sites were experimentally verified to be acetylable. Because of the lack of rigorous evidence that a site is un-acetylable, especially for the ATDS, no others but the false negative sample can be counted reasonably. Thus, only false negative rate and sensitivity are calculated for the prediction performance here, with 0% and 100% in the prediction results with high cutoff, respectively.

#### Proof

Given a tree *T* = *(V,E)*, in the complete linkage method, *D(r,s)* is computed as *D(r,s)* = Max { *d(i,j)* : Where object *i* is in cluster *r* ⊆ *V* and object *j* is cluster *s* ⊆ *V* }, At each stage of hierarchical clustering, the clusters *r* and *s*, for which *D(r,s)* is minimum, are merged.

1. Case 1(within-subclass): if Vector *i* and *j* are in a same sub-class at a height h, adding a any new vertex *k* will change the value of *d(i,j)* when *d(i,k)* <*d(i,j)* <*d(k,j),* then *d(i,j)* will be increased by *d(k,j)-d(i,j),* and the given height *h* may divide *i* and *j* to different sub-class.

2. Case 2 (between-subclass): if vertex *i* and *j* belongs to different sub-class *r* and s at a height *h,* respectively, without loss of generality, a new vector *k* is added to sub-class *r*. Then, the distance between the new set {*r, k*} and *s* is no less than distance between *r* and *s*, *D*({*r*, *k*}, *s*) ≥ *D*(*r*, *s*)) Thus, *i* and *j* will also be cut to the different subtree at the height *h*.

In all, adding new sample will not change the existed subclass relationship, because the monotone increases cluster distance definition.

To utilize the constructed hierarchical cluster tree and perform an overall prediction, we need to transform it to a classifier with features and a decision function, and fit another cutoff. According to the above hierarchical cluster results, 12 sub-classes with at least 2 peptides with the same label, 12 features were set to be the *D*(*p_i_*, *s*′*_j_*), where the function D is defined as complete linkage methods, *s*′*_j_*) represents for *j* ∈ {1,...,12} th of the 12 sub-classes, and decision function is defined as

*x* = *αmin*(*D*(*x*, *s*′*_j_*) – *βmin*(*D*(*x*, *s*′*_i_*) – *I*

where *α, β* represent for the weight of sub-class distances, and can be treated as the confidential level of the acetylability, *i* ⊂ {Acetylable subclasse} and *j* ⊂ {Un-acacylabl subclasse}, *I* for the intercept.

With the max-min distance based classifier, the 266 peptides, generated by histone filtering and 12 flanking residues window sliding processes from both human and Arabidopsis, were predicted. Among them, eighty-one have been experimentally identified as acetylation sites. Among all the remained 185 unknown lysines, almost 70% lysines were predicted to be acetylable. Of the 185, nineteen have been regarded as unacetylated based on the information we collected. For the 19 negative labeled in Arabidopsis, H3K56 and H3K79 were relabeled as acetylable-positive, and almost 50 percent unaceletalyted sites in human histones were also relabeled to be positive ones. Sensitivity and false negative rate of our prediction were estimated to be 98.4% and 1.16% based on the 81 peptides, respectively. Although the flexible cutoff may contain some false positive acetylation site, the high sensitivity should be more attractive to make the prediction result to be a useful candidate set.

### Peptide based feature conservation between histones

#### Peptide based feature conservation between Histones

In the resulted hierarchical cluster tree, all the clustered peptides happened to belong to the same histone, we chose the information gain as a statistics index, which is a monotone function to the chaos of the sub-classes. A permutation of the information gain against the null hypothesis is given by p value less than 10e - 5, where the null hypothesis is that all the histone types are random to be categorized in the different sub-classes and a highly significant correlation between histone types and the sub-classes.

### Structure factors contribution in acetylation prediction

#### 3D location of lysines are related to acetylability

**Figure 1:**
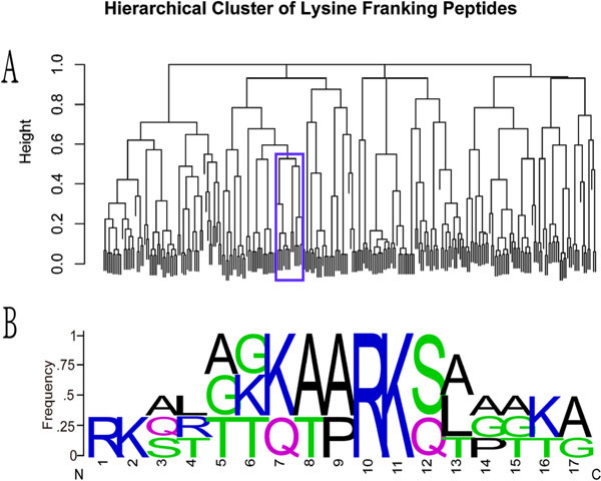
**Hierarchical Cluster of Lysine Franking Peptides and Cluster peptide Pattern** Graph A represents the hierarchical cluster dendrogram of all the peptides of human and A. thaliana. The subtree in blue rectangle contains H3K4, H3K9, H3K14, H3K18, H3K23 and H3K27, and graph B is the related sequence pattern in WebLog.

**Figure 2:**
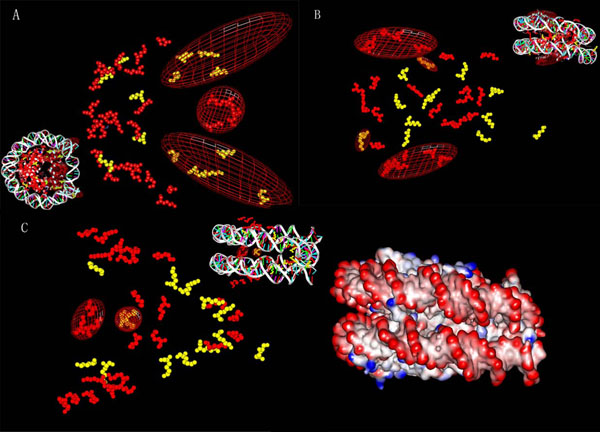
**3D location of acetylability related lysine groups.** The view angles are labeled as A, B and C. The balls in yellow represent the acetylable lysine, while red for the un-acetylable in training set. The wired ellipsoids are 3D regions related to acetylability. The lysine only and whole nucleosome parts are contained in all three views. An additional surface colored by electronically potential is added to C on the right side.

According to the constructed hierarchical cluster, in which the adjacent lysines are combined into one peptide, the clustered lysine shows not only the similarity in acetylability, but also closeness in protein location, which are not caused by the overlapping area of peptide. As in the N-terminus of the H3.1, all the lysines, H3K4, H3K9, H3K14, H3K18, H3K23 and H3K27, are acetylable and clustered together with the similar peptide pattern (Figure 1, B). To confirm the result, a permutation test was preformed against the distance of lysine location, where the null hypothesis is that all the location distances are no larger than the observed ones with the different sub-class, the test showed that a high significance of the adjacency is resulted by p value less than 10e- 5. Although the extreme N-terminuses of the core histones are not available in many crystal structure data, obviously, acetylability is associated with nucleosome location of lysines in 3D viewer (Figure 2).

Intuitionally, in the vertical viewer of the nucleosome 3D structure (2CV5) obtained from PDB [13], H3K37, H3K56, H4K31, H4K44, H4K59 (group1, Figure 2 A) are acetylable closer to the boundary regions of the DNA-binding of a nucleosome, which obviously contradistinguish to the H3K115 and H3K122 (group 2, Figure 2 A) which are closer to the middle regions of the DNA- binding of a nucleosome. In the horizontal viewer of the 3D structure, the lysines, H2BK40, H2BK43, H2BK105, H2BK113, H2BK117 and H2K122 (group 3, Figure 2 B), are obviously farther from the DNA than the acelytable sites, H4K77 and H4K79 (group 4, Figure 2 B) in the two double-strand region of nucleosome, while the third spacial stand-alone group, the pair of H4K91(group 5, Figure 2 C) are in the center of the nucleosome, which region is lack of un- acetylable lysines. Similarly, the pair of H2BK31, which are in the center of two double-strand regions of the nucleosome, have not been evaluated and therefore was not defined to be a feature group in the following prediction.

#### Other structure factors contribution in acetylation prediction

Many other structure factors have been considered to be possible features in acetylation prediction (additional file 1), but only a few have been selected. A typical type of factor is accessible surface area (ASA), which contains Percent ASA, Residue ASA, Sidechain ASA and Atom ASA. We tested the correlation coefficient between all of these values and acetylation statues by Wilcoxon Rank Sum and Signed Rank Test. Consistent with previously published results, none of them are significant at 95 percent confidential level.

### Peptide sequence and 3D structure integrated classifier

#### Construction of acetylability associated location region

To identify the distance between a lysine and acetylability associated location region, the surface of the region should be constructed properly. Here, the surface was chosen to have a shape of ellipsoid, which is fitted by the 3D location of ε-N (additional file 2),

*x*^T^*Ax* = 1

where *A* is a symmetric positive definite matrix and *x* is a vector. In such case, the eigenvectors of *A* define the principal directions of the ellipsoid and the inverse of the square root of the eigenvalues is the corresponding equatorial radii, and then the distance was calculated numerically by the simulated points of the surface.

In an Arabidopsis nucleosome, the distance between any two lysines in all histones is calculated by mapping the short peptides to protein sequence of the 2CV5 crystal structure [13].

#### Classifier construction and performance

Because of the semi-reliability of the un-acetyability, it is doubtful to train a classifier by both positive and negative sets, which are supposed to be totally correct. Under the semi-supervised situation, we adopted a hierarchy process to predict the acetyablility of Arabidopsis histones. The first hierarchy decision was done by the higher level cutoff based on peptide similarity. The lysines not contained in the predicted result were predicted by the lower level peptide-based cutoff, in which the false negative rate is shown to be 1.16%. Then the structure location was adopted to adjust the positive lysines generated in the second hierarchy. The decision strategy in the third hierarchy was the same as that used in the above peptide-based method and the distance was defined by ε-N spacial location.

Through such hierarchy process, the performances for histone acetylation prediction in Arabidopsis were 0.73, 1, 0.26 and 0 for specificity, sensitivity, false negative rate and false positive rate, respectively. Comparing to the peptide-based prediction, the previous mispredicted acetylable lysines were corrected by the structure location. Because of the existence of non- observed acetylable site, the specificity is relative lower and less reliable. There are 227 Arabidopsis peptides in the 266 selected peptides from both human and Arabidopsis, and all the 227 Arabidopsis lysine peptides were predicted based on the model generated from the rest known human histone lysines' acetylation patterns. For all the 227 Arabidopsis lysines used in prediction, there are 72.7 percent lysines are acetylable (additional file 3), which indicate that many currently unobserved acetylated status and patterns could happen under those untested special tempo-spatial conditions.

## Discussion

Histone acetylation plays a crucial role in gene expression regulation [1], and it is quite important to identify sites of acetylation in the related histons for function study and gene expression regulation. However, there are still less systemic analysis on *A. thaliana* and the widely used MS approach has limited detection and sensitivity capabilities and cannot recognize peptides that are acetylated at only low abundance. Furthermore, lysines may be modified only in some special environmental conditions, cell cycle stages, and cell types [14]. Thus, we perform a relative complete exploration on the factors that affect the acetylability of lysines in histone, and predict the acetylability of all the histone lysine in *A. thaliana*.

Almost, all the of the previously prediction methods are based on the flanking peptide sequence, and regard the structure factor as additional or less-sense features. Whereas, it is well accepted that the transcriptional effect of acetylation relies on the modification of related chromatin structures. Besides the surface accessibility, we firstly focused on the relative position of lysine in nucleosome level, and defined a new structure feature and later on adopted it to improve the predicted performance.

Beside used in the acetylation prediction, these features show certain new clues for the function mechanism at structure level, although the nucleosome structure may be dynamic in different cross-talk PTMs states. In the nucleosome crystal structure, the acetylability seems to be correlated to the boundary of DNA binding site and the interspace of the symmetrical octamer.

Along with accumulation of crystal structure, new acetylable lysines and the mechanism of histone acetylation and acetylation effects, both the new structure feature and predicted results can be verified and utilized more deeply.

## Conclusion

According a systemic exploration, we firstly focused on the relative position of lysine in nucleosome level, found a new spacial correlated acetylation factor, and then defined a ε-N spacial location based feature, which contains five core spacial ellipsoid wired areas. By incorporating the new feature, the performance of predicting the acetylability of all the histone lysines in *A. Thaliana* was promoted, in which the previous mispredicted acetylable lysines were corrected by comparing to the peptide-based prediction.

## Materials and Methods

### Data collection

We searched published literature in PubMed with the key word 'arabidopsis AND histone AND (acetylated OR acetylation OR acetyltransferase OR acetylate)', and 110 items returned by Dec. 2009. All the acetylation sites and acetyltransferases were retrieved manually.

The two histone H3 variants, H3.1 and H3.2, which are differing in the abundance of silencing and activating chromatin [15], are both considered here.

The Arabidopsis genome has 11 H2B genes, which encode proteins that differ mainly in the amino acid sequence of their N-terminal tails. Here, we selected H2B.6, H2B.7 and H2B.10, with experimentally acetylation evidence[9]. There are 13, 12, 12, 22, 16, 21 lysines in Histone H2A.1, H2A.2, H2A.3, H2A.4, H2A.5 and H2A.6, respectively, only two sites, K5 and K144H2A, have been verified for acetylation [8].

#### Histone filter and peptide generation

Only the proteins with identified acetylated sites were considered here, from which the classified peptides were retrieved.

All the peptides are generated by using a sliding window of a maximum number of 12 residues flanking each lysine in the filtered histones.

#### Sequence logos

Sequence Logos. Sequence logos were created by WebLogo [16]

#### Accessible Surface Area

All the Accessible surface area(ASA), which contains Percent ASA, Residue ASA, Sidechain ASA and Atom ASA, were calculated by Surface Racer, where the Van del Waals radii sets is Richards 1977 and probe radius is 1.2 in Angstroms [17]

## Competing interests

The authors declare that they have no competing interests.

## Acknowledgements

This study was supported by State Key Program of Basic Research of China (Grant 2007CB108800, 2009CB918402, 2010CB912102); National Natural Science Foundation of China (Grant 30870575), and Science and Technology Commission of Shanghai Municipality (06DZ22923). Publication of this supplement was made possible with support from the International Society of Intelligent Biological Medicine (ISIBM).

This article has been published as part of *BMC Genomics* Volume 11 Supplement 2, 2010: Proceedings of the 2009 International Conference on Bioinformatics & Computational Biology (BioComp 2009). The full contents of the supplement are available online at http://www.biomedcentral.com/1471-2164/11?issue=S2.

## Supplementary Material

Additional file 1 All the lysines associated 3D structure factor Click here for file

Additional file 2 Epsilon N 3d Location and Atom ASA Click here for file

Additional file 3 **All the prediction results.** All the IDs are named as database name, ID, Protein name. All the lysines in the training set are labeled with P or N in the end. Click here for file

Additional file 4 All the training and testing peptides Click here for file
